# cAMP Response Element-Binding Protein (CREB): A Possible Signaling Molecule Link in the Pathophysiology of Schizophrenia

**DOI:** 10.3389/fnmol.2018.00255

**Published:** 2018-08-30

**Authors:** Haitao Wang, Jiangping Xu, Philip Lazarovici, Remi Quirion, Wenhua Zheng

**Affiliations:** ^1^Department of Neuropharmacology and Drug Discovery, School of Pharmaceutical Sciences, Southern Medical University, Guangzhou, China; ^2^School of Pharmacy Institute for Drug Research, Faculty of Medicine, The Hebrew University of Jerusalem, Jerusalem, Israel; ^3^Douglas Mental Health University Institute, McGill University, Montreal, QC, Canada; ^4^Faculty of Health Sciences, University of Macau, Taipa, China

**Keywords:** CREB, schizophrenia, neurotransmitter, neurodevelopment, dopamine

## Abstract

Dopamine is a brain neurotransmitter involved in the pathology of schizophrenia. The dopamine hypothesis states that, in schizophrenia, dopaminergic signal transduction is hyperactive. The cAMP-response element binding protein (CREB) is an intracellular protein that regulates the expression of genes that are important in dopaminergic neurons. Dopamine affects the phosphorylation of CREB via G protein-coupled receptors. Neurotrophins, such as brain derived growth factor (BDNF), are critical regulators during neurodevelopment and synaptic plasticity. The CREB is one of the major regulators of neurotrophin responses since phosphorylated CREB binds to a specific sequence in the promoter of BDNF and regulates its transcription. Moreover, susceptibility genes associated with schizophrenia also target and stimulate the activity of CREB. Abnormalities of CREB expression is observed in the brain of individuals suffering from schizophrenia, and two variants (-933T to C and -413G to A) were found only in schizophrenic patients. The CREB was also involved in the therapy of animal models of schizophrenia. Collectively, these findings suggest a link between CREB and the pathophysiology of schizophrenia. This review provides an overview of CREB structure, expression, and biological functions in the brain and its interaction with dopamine signaling, neurotrophins, and susceptibility genes for schizophrenia. Animal models in which CREB function is modulated, by either overexpression of the protein or knocked down through gene deletion/mutation, implicating CREB in schizophrenia and antipsychotic drugs efficacy are also discussed. Targeting research and drug development on CREB could potentially accelerate the development of novel medications against schizophrenia.

## Introduction

The cAMP-response element binding protein (CREB) is localized in the nucleus and acts as a transcription factor, which binds to the cAMP response element (CRE) of the promoters of its target genes, upon phosphorylation at Ser133 by different receptor-activated protein kinases, such as protein kinase A (PKA), calmodulin-dependent protein kinase (CaMK), mitogen-activated protein kinases (MAPK), and other kinases (Alberini, 2009). Once CREB is activated and CREB-binding protein (CBP) is recruited, transcription is initiated (Dyson and Wright, 2016). The activity of CREB in neurons has been correlated with various intracellular processes, including proliferation, differentiation, survival, long-term synaptic potentiation, neurogenesis, and neuronal plasticity (Alberini, 2009; Landeira et al., 2016; Kitagawa et al., 2017). Recent studies propose that CREB is involved in signaling pathways leading to pathogenesis and therapy of certain mental disorders, including schizophrenia, making CREB an important focus of investigation (Ren et al., 2014).

Schizophrenia is a severe mental illness that changes a patient’s way of thinking, feeling, and social behavior (Adan et al., 2017). The onset of typical symptoms usually occurs around puberty or early adulthood (van Rijn et al., 2011). This phenomenon is assumed to be controlled by hormones and a latent immune vulnerability (Walker et al., 2008; Kinney et al., 2010). The current hypothesis of schizophrenia claims that the positive symptoms of the disease are linked to hyperactive dopaminergic activity, mediated by D2 dopamine receptors (D2R) in subcortical brain regions such as the striatum and the nucleus accumbens (Toda and Abi-Dargham, 2007; Lindenmayer et al., 2013), while the deficits in dopamine activity mediated by D1 dopamine receptors (D1R) are responsible for the negative symptoms and cognitive impairment (Toda and Abi-Dargham, 2007; Lindenmayer et al., 2013). Schizophrenia is also considered as a neurodevelopmental disorder, and this is confirmed by epidemiological, developmental, and neuroimaging studies (Owen et al., 2011). Moreover, patient population genetics suggest that schizophrenia may result from a combination of genetic factors and environmental insults, including prenatal infection, perinatal complications, and drug abuse (Stepniak et al., 2014).

The following evidences propose CREB as a convergent dopaminergic signaling protein in schizophrenia: (1) *in vitro* and animal studies show that dopamine receptor signaling increases the phosphorylation of CREB (Lukasiewicz et al., 2016). Activated CREB promotes the expression of brain-derived neurotrophic factor (BDNF) (Yoo et al., 2017), while on the other hand, BDNF promotes the activation of CREB through tropomyosin receptor kinase (Trk) B receptors (Finkbeiner et al., 1997). Direct relationships were also found between antipsychotic drugs binding to D2R, therapeutic effect, and stimulation of CREB phosphorylation *in vitro* and in animal models (Konradi et al., 1993; Lee et al., 2010). (2) Data from schizophrenic patients also support the interaction between CREB and BDNF (Palomino et al., 2006; Tejeda and Diaz-Guerra, 2017). As a downstream target of BDNF, protein kinase B (Akt), glycogen synthase kinase 3β (GSK3β), disrupted-in-schizophrenia-protein 1 (DISC1), neuregulin-1 (NRG-1), and dysbindin-1 are common associated susceptibility genes (Zheng et al., 2012). Interestingly, CREB is a substrate for Akt and GSK3β phosphorylation: Akt at Ser133 (Li et al., 2011), while GSK3β at Ser129 (Horike et al., 2008); and (3) besides these studies that implicate CREB in schizophrenia, evidence is provided by patients’ postmortem pathological studies. The protein and gene levels of CREB and the binding activity of CREB to CRE in schizophrenic brains were significantly decreased in the cingulate gyrus (Yuan et al., 2010; Ren et al., 2014), an integral brain limbic system structure, which is involved with emotion, learning, and memory and found to be smaller and with lower neural activity in people with schizophrenia. Therefore, the CREB pathway may represent a promising target for the development of innovative interventions for schizophrenia. In the current review, the role of the CREB signaling pathway in the pathophysiology of schizophrenia will be discussed.

## Molecular Structure of CREB

The CREB genes in both mouse and human consist of 11 exons, and 3 isoforms designated α, β, and Δ are produced through alternative splicing (Ichiki, 2006). These isoforms, expressed in most tissues, are identical in function. Primary structure studies show that the full-length sequence of CREB could be divided into four functional domains from the N-terminus to C-terminus, namely (i) Q1 basal transcriptional activity domain; (ii) kinase inducible domain (KID); (iii) a glutamine-rich, Q2 domain for constitutive activation; and iv) a basic region/leucine zipper domain (bZIP) forming a homodimer and responsible for binding to DNA (Xu et al., 2007). The Q1 domain localizes at the N-terminus of CREB, interacts with TATA binding protein, and promotes gene transcription (Felinski and Quinn, 2001). The KID is located in the middle region; central to this region is Ser133 and the phosphorylation of Ser133 by multiple protein kinases is necessary for the activation of CREB (Sun et al., 2016). The upstream protein kinases activating CREB include PKA, Akt, protein kinase C (PKC), calcium/calmodulin-dependent protein kinase II (CaMKII), p90 ribosomal S6 kinase (p90RSK), casein kinase I, and casein kinase II (Wen et al., 2010; Trinh et al., 2013). The Q2 domain is responsible for binding with RNA polymerase II initiation complex. This domain is responsible for the recognition and binding to the canonical CRE, 5′-TGACGTCA-3′ (Altarejos and Montminy, 2011). The carboxy terminal of CREB is a bZIP dimerization domain, which is required for the dimerization of CREB (Schumacher et al., 2000). The Mg^2+^ ions facilitate the binding activity of bZIP to CRE by more than 25-fold (Schumacher et al., 2000). As mentioned earlier, CREB could be phosphorylated by PKA at Ser133; however, PKA phosphorylation does not alter the secondary structure of CREB, and, therefore, has no effect on the binding of CREB to DNA (Richards et al., 1996). Besides CREB, cAMP response element modulator (CREM) and activating transcription factor-1 (ATF-1) are also members of the CREB family. The structure and biological functions of both CREM and ATF-1 are similar to CREB, which forms heterodimers with ATF-1 or CREM (Wu et al., 1998). However, the interactions of CREB/CREM and CREB/ATF-1 in the pathophysiology of schizophrenia are unknown and will not be further addressed.

### Dopamine-Mediated Signaling Affects the Activity of CREB

The CREB is a critical molecule involved in the signal transduction of dopamine receptors. Binding of dopamine to its receptors enhances the phosphorylation of CREB through multiple pathways: (i) binding of dopamine to D1R elevates intracellular cAMP levels and activates PKA followed by the phosphorylation of CREB (Chartoff et al., 2003; Boyd and Mailman, 2012); (ii) binding of dopamine to D2R reduces cAMP production and adenylate cyclase activity followed by reduced phosphorylation of CREB (Boyd and Mailman, 2012). However, repeated treatment with the selective D2R agonist, such as quinpirole, enhances PKA activity and increases phospho-CREB expression in the nucleus accumbens (Culm et al., 2004). Importantly, CREB activation in the nucleus accumbens attenuates prepulse inhibition (PPI) disruption (Culm et al., 2004). Thus, chronic administration of D2R agonist promotes the phosphorylation of CREB, and the mechanism might be related to downregulation of D2R receptors; (iii) D2R is also coupled to phospholipase Cβ (PLCβ). Activation of D2R by its agonist quinpirole causes an elevation of intracellular Ca^2+^ and activation of PKC. The Ca^2+^/CaMK and PKC are the upstream protein kinases phosphorylating CREB and, therefore, enhanced Ca^2+^ and activation of PKC cause the phosphorylation and activation of CREB (Yan et al., 1999). Dopamine- and cAMP-regulated phosphoprotein of molecular weight 32 kDa (DARPP-32) is possibly a molecule that links D2R-mediated signaling and CREB. In DARPP-32 knockout mice, the basal phosphorylation levels of CREB were elevated, and the ability of D2R to induce phosphorylation of CREB was lost (Yan et al., 1999); (iv) Stimulation of D1R and D2R with agonists activates Akt kinase, which translocates to the nucleus. Activated Akt directly phosphorylates CREB at Ser133 in striatal neurons (Brami-Cherrier et al., 2002); (v) CREB stimulates the expression of a number of genes containing CREs (5′-TGACGTCA-3′) in their promoter regions (Montminy, 1997), which may be associated with schizophrenia such as D1R, serotonin transporter, and synapsin 1 (Meyer et al., 1993; Montminy, 1997). It has been also reported that D1R/D2R plays a synergistic role in inducing CREB–DNA binding activities (Kashihara et al., 1999).

### Signaling Cascades Regulating the Activity of CREB

**FIGURE 1:**
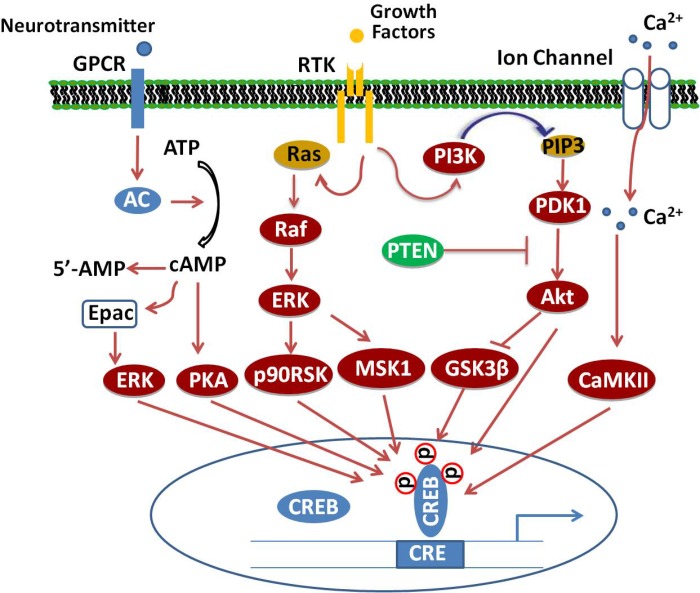
Signaling cascade of CREB. Adenylate cyclase (AC) activated upon stimulation of cellular G-protein-coupled receptors (GPCR) by neurotransmitters increases cAMP levels, which, in turn, activate PKA. The catalytic subunits of PKA translocate into the nucleus and phosphorylate CREB at Ser133. Binding of growth factors to receptor tyrosine kinases (RTK) stimulate the activation of PI3K/Akt/GSK3β, Ras/Raf/MEK/ERK/p90/RSK and ERK/MSK1 signaling pathways, which subsequently enhance the phosphorylation of CREB at different sites. Additionally, activation of excitatory NMDA receptors will increase the phosphorylation of CREB through Ca^2+^/CaMK-dependent pathways.

Several different protein kinases phosphorylate CREB, making it a convergent target for multiple intracellular signaling cascades. The most important posttranscriptional modification is the phosphorylation of the amino acid residue Ser133 in the KID domain (Guo et al., 2017). This domain contains multiple phosphorylation sites for many canonical signaling pathways, such as Ras/Raf/MAPK/p90RSK, Ca^2+^/CaMK, PI3K/Akt/GSK3β, and cAMP/PKA pathways (Tang et al., 2014; Cheng et al., 2016; Xi et al., 2016; Zeng et al., 2016; Heckman et al., 2018), which, upon activation, lead to CREB’s antagonistic effects (**Figure 1**).

Both cAMP and cGMP show regulatory functions in mental disorders (Ben Aissa et al., 2016) and both of them could regulate the phosphorylation of CREB. It is well documented that cAMP, through PKA, stimulates the phosphorylation of CREB at Ser133 and causes the activation of CREB (Herold et al., 2011; Heckman et al., 2018), while cGMP activates the downstream protein cGMP-dependent protein kinase G (PKG), which also phosphorylates the transcription factor CREB at Ser133 (Lueptow et al., 2016). This dual phosphorylation by PKA and PKG may amplify the CREB activity (Lu et al., 1999).

In addition to the canonical cAMP–PKA–CREB pathway, the exchange protein directly activated by cAMP (Epac) is another cAMP-binding effector protein. *In vitro*, binding affinities of cAMP for PKA and Epac are similar (Bos, 2006). cAMP activates the small GTP binding protein Rap1 through Epac and activates extracellular signal-regulated kinase 1/2 (ERK1/2) signaling, which subsequently leads to the phosphorylation of CREB. The cAMP–Epac–ERK–CREB signaling pathway is known to mediate neurotrophic and neuroprotective functions (Grimes et al., 2015).

**FIGURE 2:**
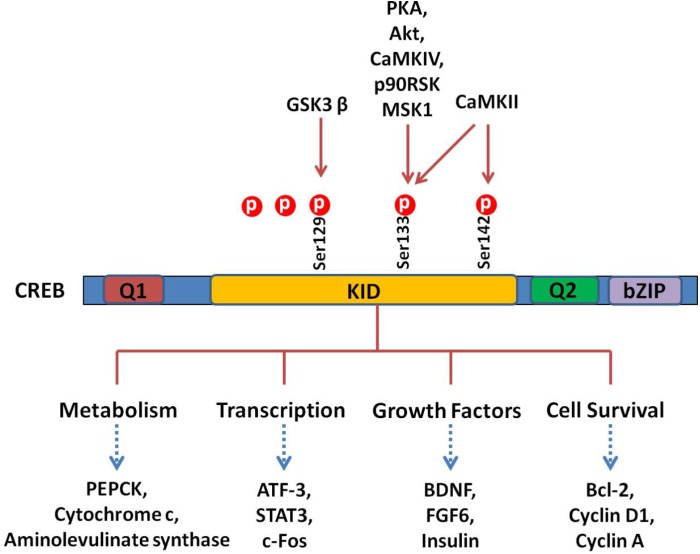
CREB and its downstream substrates. The CREB contains Q1, kinase-inducible domain (KID), Q2, and bZIP domains. The crucial event in the activation of CREB is the phosphorylation of Ser133 in KID. This domain could be phoshphorylated by multiple protein kinases such as PKA, Akt, CaMKs, p90RSK, and MSK1. The CREB as a nuclear transcription factor binds to CRE (cAMP response element), regulating transcription activity of its downstream substrates, which regulate neuronal processes, including metabolism and survival and expression of different transcription factors and growth factors.

Cells treated with phosphodiesterase inhibitors will also prevent the degradation and increase of the endogenous level of cAMP (Guo et al., 2017). The activated PKA enters into the nucleus and promotes the binding of phosphorylated CREB to a CRE region (5′-TGACGTCA-3′). The CBP is subsequently recruited and bound to CREB, which co-activates CREB. Activation of CREB consequently stimulates or inhibits the expression of its downstream target genes, including genes involved in metabolism (such as *PEPCK, cytochrome c*, and *aminolevulinate synthase* ), transcription (such as *ATF-3, STAT3*, and *c-fos* ), cell survival/cell cycle (such as *bcl-2, cyclin D1*, and *cyclin A*), and growth factors (such as *BDNF* and *FGF6*) (Sakamoto and Frank, 2009). A schematic survey of target genes regulated by CREB is presented in **Figure 2**. Although CREB could bind to many regulatory gene regions, it may not be critical for the expression of these genes. The most plausible explanation claims that the gene is under regulation by more than one transcription factor and coactivator and, therefore, deletion of one transcription factor is not always effective in the final gene expression (Lemberger et al., 2008).

There are three phosphorylation sites existing in the amino-terminal of CREB, and the phosphorylation of Ser133 is the most studied. The phosphorylation of Ser133 leads to a 10- to 20-fold increase in CREB’s transcriptional activity (Mayr and Montminy, 2001). Functionally, phosphorylation of this site promotes the transcription and expression of CREB target genes (Delghandi et al., 2005). Notably, phosphorylation of Ser133 mainly influences the activity of the trans-activation domain, while it has no effect on the affinity of CRBE to the CRE sequence. Since mutation of Ser133 destroys the transcription activity of this protein (Karin and Smeal, 1992), phosphorylation of Ser133 activates CREB’s trans-activation domain through changing its conformation, resulting in better interaction with other coactivators (Karin and Smeal, 1992; Mayr and Montminy, 2001). It is hypothesized that phosphorylation of CREB makes the trans-activation domain closer to the amino terminal, which interacts with CREB dimers or some other DNA-binding protein(s) (Mayr and Montminy, 2001). The CREB can be phosphorylated at other amino acids than Ser133, including Ser129, Ser142, and Ser143 (Shaywitz and Greenberg, 1999). Different kinases such as CaMK IV phosphorylate CREB at Ser133 only, while CaMK II phosphorylates CREB at both Ser133 and Ser142. Phosphorylation of Ser142 by CaMK II inhibits CREB (Sun et al., 1994), while phosphorylation of Ser133 enhances the CREB trans-activation dramatically. These results indicate that cAMP is a positive stimulator of transcription. Different evidences support the hypothesis that phosphorylation of CREB at Ser142/143 blocks the phosphorylation of CREB at Ser133 and attenuates the binding of CBP to CREB (West et al., 2002).

Phosphatase enzymes, which remove phosphate groups from protein substrates phosphorylated by kinases, are essential to many neuronal functions, because phosphorylation and dephosphorylation reactions serve diverse roles in regulatory signaling networks. In mammals, serine/threonine protein phosphatases include PP1, PP2A, PP2B, and PP2C (Shi, 2009). All of these phosphatases act on CREB and promote the dephosphorylation of CREB. The PP2A in the nucleus is the most effective phosphatase that dephosphorylates CREB (Wadzinski et al., 1993). Specifically, PP2A efficiently dephosphorylates PKA-stimulated CREB phosphorylation and, therefore, attenuates cAMP or PKA-stimulated gene transcription (Wadzinski et al., 1993). Dephosphorylation of Ser133 residue by PP1 also represses CREB activity (Yang et al., 2015) and this effect is involved in long-term depression at glutamatergic synapses (Mauna et al., 2011). The PP2B, a calcium-dependent phosphatase, is also involved in accelerating the decay of phosphorylated CREB at Ser133 (Bito et al., 1996). The PP2B may act on CREB indirectly and the possible target for PP2B is PP1. Synaptic Ca^2+^ entry triggers the activation of Ca^2+^/calmodulin signaling and subsequently causes phosphorylation of PP2B, which acts as an inhibitory subunit of PP1 (Bito et al., 1996). The role of PP2C on the dephosphorylation of CREB is not yet clear. Additional proteins such as phosphatase and tensin homolog (PTEN) contribute to the dephosphorylation of CREB. It has been confirmed that CREB is a substrate of PTEN in the nucleus where it is colocalized with phosphorylated CREB and directly dephosphorylates CREB at Ser133 (Gu et al., 2011). The duration and extent of CREB phosphorylation are parallel with the transcriptional regulation of target genes containing the CRE sequence (Hagiwara et al., 1992). The balance between kinases responsible for phosphorylating CREB and phosphatases capable of dephosphorylating CREB determines the degree of CRE-dependent gene transcription (Hagiwara et al., 1992).

### CREB-Mediated Transcription in the Nucleus Accumbens (NAc) in Mood Regulation

The CREB is expressed throughout the striatal regions, including the nucleus accumbens (NAc) (Mantamadiotis et al., 2002). The activity of CREB in the NAc is a pivotal modulator of an animal’s behavioral phenotype response to psychiatric stimuli (Barrot et al., 2002). Upon exposure to stress, CREB is stimulated in the NAc, and both D1R- and D2R dopaminergic neurons are involved in the phosphorylation of CREB (Blendy, 2006). Besides dopaminergic neurons, the biological function of CREB in the NAc is also regulated by glutamatergic inputs (Dudman et al., 2003). Current studies indicate that alterations in CREB activity within the NAc represent an important gate between emotional stimuli and animal behavioral response (Barrot et al., 2002). Experimental changes in CREB activity in the NAc are sufficient to change animal behavior. For instance, elevations of CREB activity through viral-mediated gene transfer of CREB within the NAc reduces sensitivity to emotional and stress stimuli (Barrot et al., 2002). Similarly, a sustained activation of CREB in the NAc produces anhedonia-like symptoms, and pro-depression-like symptoms in both acute and chronic stress-induced depression animal models in rats or mice (Pliakas et al., 2001; Newton et al., 2002). Conversely, dominant negative mutant CREB (mCREB) in the rat NAc enhanced cocaine, morphine, and sucrose preference (Barrot et al., 2002; Dinieri et al., 2009). These data demonstrate that CREB activity in the NAc is highly related with drug withdrawal, depression, and other dysphoric states. Additionally, CREB in the NAc also affects anxiety-like behavior in animals. Loss of function of CREB within the NAc produces anxiety-like effects, whereas elevations of CREB function leads to the opposite phenotype (Barrot et al., 2002; Wallace et al., 2009). These studies raise the possibility that modulation of CREB in the NAc contributes to the development of different mood disorders (Carlezon et al., 2005). In animal models of schizophrenia, PKA activity and CREB phosphorylation in the NAc are decreased, and treatment with antipsychotics increases CREB activity in the NAc, and this neuro-adaptive response facilitates the recovery of sensorimotor gating (Culm et al., 2004), which is seriously disrupted in schizophrenic patients. As a transcription factor, activated CREB regulates the transcription of various genes including tyrosine hydroxylase, serotonin 2A receptors, and other genes possibly implicated in schizophrenia (Culm et al., 2004). The CREB phosphorylation in the NAc, after treatment with quinpirole, is proposed to mediate gene transcription and subsequently promoting the recovery of sensorimotor gating (Culm et al., 2004). These studies emphasize on the important role of the target genes of CREB, especially genes specifically expressed in the brain region related to schizophrenia. The CREB and such target genes could be utilized, therefore, in drug discovery efforts.

### Interaction Between CREB and Neurotrophins-Mediated Signaling

The “neurodevelopmental hypothesis of schizophrenia” emphasizes that the abnormalities of early brain development increase the risk for the subsequent emergence of clinical symptoms (Schmidt and Mirnics, 2015). In this hypothesis, schizophrenia is associated with a subtle brain lesion that is caused by a combination of genetic and/or early environmental factors and that eventually interacts with normal maturational processes of the synapse and brain, to facilitate symptoms such as psychosis (Schmidt and Mirnics, 2015).

The BDNF is an important neurotrophin that promotes the development of certain populations of neuronal cells and confers neuroprotection under different conditions (Nieto et al., 2013). Dysfunction of BDNF signaling leads to deficits in neuronal growth and synaptic transmission, leading to disorganized brain function, which contributes to the development of schizophrenia (Palomino et al., 2006). Mutual relationships between BDNF and CREB are well documented: BDNF promotes the phosphorylation of CREB, which, in turn, promotes the transcription of BDNF gene. Treatment of neurons with BDNF triggers CREB phosphorylation (Pizzorusso et al., 2000). The BDNF activates CREB, in part, by increasing intracellular Ca^2+^ that leads to the activation of CaMKIV, which phosphorylates CREB. Neuronal exposure to BDNF also activates the Ras/Erk/Rsk pathway that causes CREB phosphorylation (Finkbeiner et al., 1997). The pathway of BDNF-induced CREB phosphorylation involves BDNF receptor TrkB stimulation, because treatment with the pan Trk antagonist, K252a, completely blocked CREB phosphorylation (Pizzorusso et al., 2000). Neuronal dendritic growth is essential for the neuronal network’s electrophysiological activity, and the dendritic development is impaired in schizophrenia (Glausier and Lewis, 2013). Among different neurotrophins that regulate the development of dendrites, BDNF regulates the dendritic length and complexity of MAPK and CREB signaling (Finsterwald et al., 2010). Moreover, phosphorylation of TrkB induces activation and translocation of MAPKs from the cytoplasm into the nucleus, which subsequently causes the activation of nuclear kinase mitogen- and stress-activated kinase (MSK 1) (Deak et al., 1998). The MSK1 is a protein kinase expressed in the central nervous system (Arthur et al., 2004) and a major CREB (Ser133) kinase that is direct downstream target of BDNF receptors and the MAPK cascade (Daumas et al., 2017). Indeed, MSK1^-/-^ mice displayed deficits in experience-dependent synaptic plasticity (Correa et al., 2012), spatial and recognition memory tasks, and in BDNF-mediated phosphorylation of CREB (Arthur et al., 2004; Karelina et al., 2012). Additionally, BDNF stimulates the binding of CREB to the promoter region of cypin, which is a major PSD-95-binding protein. Enhanced transcription of cypin results in an increase of dendrite branching (Kwon et al., 2011). Maternal inflammation during pregnancy affects the neuronal cell survival in the offspring due to inflammatory factors, which induce axonal loss. The BDNF is a potent pro-survival factor, and the neuroprotective effect of BDNF is associated with the activation of CREB signaling (Fujino et al., 2009; Mohammadi et al., 2018). Cumulatively, these findings show that BDNF plays pivotal roles in both neuronal development and survival, and importantly, CREB is a downstream molecule responsible for some of the neurobiological functions of BDNF.

Interestingly, treatment with BDNF activates CREB through TrkB receptors (Pizzorusso et al., 2000). On the contrary, the expression of BDNF is regulated by CREB (Tao et al., 1998). The PKA enhances the phosphorylation of CREB at Ser133, which leads to the nuclear localization and activation of CREB (Guo et al., 2017). The promoter region of BDNF contains CRE; activated CREB binds to CRE and promotes the transcription of BDNF (Tao et al., 1998). Upregulation of BDNF mRNA expression is parallel to increased phosphorylated CREB expression (Guo et al., 2017), and inhibition of PKA/CREB pathway attenuates the level of BDNF in neurons (Xue et al., 2016).

### Biological Functions of CREB in the Central Nervous System

The CREB has been implicated in the regulation of a variety of biological functions (Steven and Seliger, 2016). Biological functions in the brain, such as the contribution of CREB to synaptic plasticity and neurodevelopment, have also been established (Fujino et al., 2009). Immunohistochemical analyses of CREB and ATF-1 indicated that ATF-1 was expressed in trophectoderm and inner cell mass cells at E3.5 of the mice embryo (Bleckmann et al., 2002). The expression of CREB could be detected at E3.5 and its expression increased during later stages in the epiblast and cells derived thereof (Bleckmann et al., 2002). The CREB-null mice, which carry a mutation that knock down all functional isoforms of CREB gene, are smaller than their littermates and face survival problems due to respiratory distress (Rudolph et al., 1998). As CREB knockout is lethal during perinatal development, the function of CREB in the brain in adult mice cannot be investigated in CREB-null mice. To overcome this problem, conditional knockout of CREB in the brain of developing and adult mice was developed using the Cre/loxP system (Mantamadiotis et al., 2002). Absence of CREB in the central nervous system was correlated to upregulation of CREM, but with no significant pathologies. As CREB and CREM play a similar effect in cellular survival, CREM upregulation in the brain is sufficient to maintain neuronal survival. Similarly, loss of only CREB in brain has limited effect on neuronal survival. However, deletion of both CREB and CREM caused non-specific neuronal cell death and progressive neurodegeneration in the brain hippocampus and in the dorsolateral striatum (Dawson and Ginty, 2002; Mantamadiotis et al., 2002). Therefore, it is reasonable to conclude that knockdown of CREB and CREM in neurons of the developing CNS may cause apoptosis, and postnatal ablation of these genes in adulthood may result in neuronal degeneration. Cumulatively, these findings support the concept that CREB expression and transcriptional activity are regulated in both embryonic and mature brain, and it is implicated in neuronal survival as well as in neurogenesis, processes associated with the pathology of schizophrenia.

Additionally, ablation of CREB resulted in neuronal degeneration in hippocampus and striatum (Dawson and Ginty, 2002) contributing to the pathogenesis of neurodegenerative diseases and mood disorders, such as schizophrenia and depression (Wang et al., 2015). It is well established that CREB is necessary for spatial memory (Sekeres et al., 2010). As a binding protein to CREB, conditional knockout of CBP in the mice brain caused significant impairment in spatial, associative, and object-recognition memory (Chen et al., 2010). In Alzheimer’s disease (AD) transgenic mice model, TgCRND8 mice exhibited a profound impairment in the ability in spatial memory. However, increasing CREB function in the CA1 region of dorsal hippocampus rescued the spatial memory deficits (Yiu et al., 2011). In recent experiments, it has been demonstrated that CREB represents an important target for drug development in the therapy of AD (Guo et al., 2017).

Besides its role in neurodegenerative diseases, CREB is also proposed to be involved in the disease process of psychiatric disorders, such as schizophrenia (McGirr et al., 2016), autism (Lyu et al., 2016), drug addiction (Fisher et al., 2016), and depression (Zhou et al., 2016). For example, PPI deficits were observed in rats treated with dopamine D2R agonists and in individuals suffering from schizophrenia, while chronic quinpirole or ropinirole drug treatment produced sustained PPI recovery, requiring CREB activity in the nucleus accumbens of rats (Berger et al., 2011). Recombinant lentivirus LV-CREB133 expressing a dominant negative CREB decreased synapse and spine density, inhibited neurogenesis, and attenuated the expression of synapsin and spinophilin (Zhang et al., 2016). However, LV-VP16-CREB, a constitutively active CREB, increased synapse density and dendrite complexity, enhanced neurogenesis, and increased the expression of synaptic proteins (Zhang et al., 2016). This suggests that CREB is involved in the neuronal plasticity and possibly implicated in modulating schizophrenia-related behaviors. Another example is the link between CREB and autism. The CREB (alpha and delta isoforms)-deficient mice were less active and more inhibited in the behavioral assays of elevated plus maze, open field, and light/dark box (Hebda-Bauer et al., 2004), an effect similar with altered exploratory behavior in autism spectrum disorder (Veenstra-VanderWeele et al., 2012). As an important protein involved in neuronal development and synaptic plasticity, the relationship between CREB and autism is receiving increasing attention (Nuytens et al., 2013; Gao et al., 2016).

It is noteworthy that chronic CREB activation may also cause deleterious consequences. Chronic activation of CREB led to sporadic epileptic seizures and a significant loss of hippocampal neurons (Lopez de Armentia et al., 2007). Chronic enhancement of CREB activity also delayed the retrieval of spatial information (Viosca et al., 2009). Further studies indicate that the pathological consequences resulting from CREB inhibition and CREB activation are mediated through different mechanistic processes (Sakamoto et al., 2011). The CREB inhibition triggers cell death through a pro-apoptotic signal pathway (Zeng et al., 2016), while chronic CREB activation triggers loss of neurons through an excitotoxicity mechanism (Lopez de Armentia et al., 2007). This suggests that the timing of CREB regulation may be a key for the various associative changes that culminate in cellular neuronal responses.

### Interactions Between CREB and Susceptibility Genes for Schizophrenia

**FIGURE 3:**
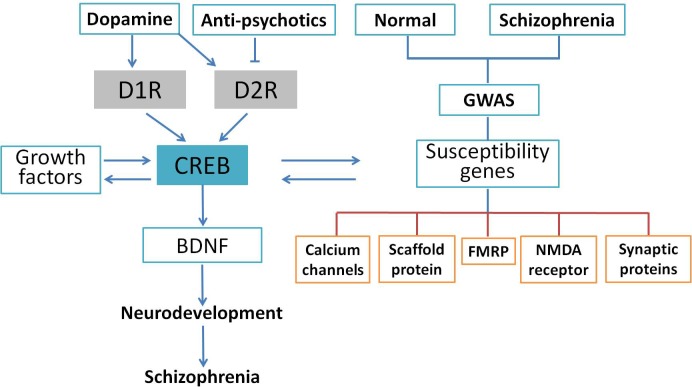
CREB as an integrative signaling molecule involved in schizophrenia. Dopamine stimulates the phosphorylation of CREB through D1R-mediated cAMP/PKA pathway or inhibits it through the D2R-mediated cAMP/PKA pathway. The CREB may also affect neurotrophins (BDNF) and other protein expressions and susceptibility genes associated with schizophrenia. The GWAS indicated that gene sets related to calcium channels, activity-regulated cytoskeleton-associated scaffold protein, FMRP, PSD-95, NMDA receptor, and synaptic proteins were potential candidates altered in the schizophrenic patients. The CREB-induced dysregulation in neuronal signaling may lead to neurodevelopmental deficits followed by schizophrenic behavior.

Schizophrenia is a complicated central nervous disease, and the causes of schizophrenia include genetic factors and gene–environment interactions. In the last decade, a number of chromosomal regions and genes have been studied with molecular biology and genetic analyses. However, there has been no consistent single gene variation confirmed with the development of this illness, and the contribution of genetic factor remains obscure at this time (Tandon et al., 2008). Genome-wide association study (GWAS) provides an unbiased assessment of variation through investigating the entire genome. Many GWAS of schizophrenia have been published in the past 10 years (Bergen and Petryshen, 2012; Visscher et al., 2017), and numerous single nucleotide polymorphisms have been found to be associated with schizophrenia (Giusti-Rodriguez and Sullivan, 2013; Hertzberg et al., 2015). Although it is too premature to link these studies to schizophrenia genetics, current available analyses support that some of the previously implicated pathways such as calcium signaling, CREB signaling, and NMDA receptors are involved in the pathology of schizophrenia. For example, an investigation of the *de novo* mutations in 623 families with schizophrenia in Bulgaria indicated that synaptic genes, such as genes encoding postsynaptic density proteins, cytoskeleton-associated scaffold proteins, and *N*-methyl-D-aspartate (NMDA) receptor, were enriched in these mutated genes (Fromer et al., 2014). These results were confirmed by another comprehensive case–control study of schizophrenic patients in Sweden using exome analysis (Purcell et al., 2014). Collectively, GWAS indicates an important role for synaptic genes and genes regulating synaptic plasticity in the risk for schizophrenia. Genetic alterations extracted from GWAS data are shown in **Figure 3**. Based on these findings, Forero et al. (2016) summarized genes involved in the cAMP/PKA/CREB signaling pathway that could be candidates for schizophrenia. It was suggested that synaptic genes, including *CREB1, CREM, PPP3CB*, and *PRKAR1A*, are involved in the etiology of schizophrenia-related psychiatric disorders and regulated by the cAMP/PKA/CREB signaling pathway (Forero et al., 2016). Genetic studies have identified many genes and pathways implicated in schizophrenia, but the genetic liability needs further verification. Using Sanger method and next-generation sequencing, a study at the genome-wide level was performed to test the single nucleotide variants of 10 traditional candidate genes in 727 patients with schizophrenia and 733 controls. Unfortunately, none of the 10 traditional candidate genes had single nucleotide variants showing an association with schizophrenia (Crowley et al., 2013). Consistently, genome-wide array comparative genomic hybridization in five large pedigrees with schizophrenia showed that no linkage exist between any copy number variant and schizophrenia (Timms et al., 2013). In summary, exome studies have indicated that rare *de novo* and transmitted mutations contribute to the development of schizophrenia. However, it is worthy of our attention that so far there has been no significant association with a gene. The penetrance of *de novo* mutation to chromatin regulation is yet unknown and deserves further clarification.

With this background, we would like to address a few studies focusing on NRG-1 and the DISC-1 and dysbindin-1 genes in relation to schizophrenia. These genes play a role in both neural signaling and development, and are associated with schizophrenia (Gong et al., 2014; Mostaid et al., 2017; Prats et al., 2017). It would be interesting to determine the interaction between CREB and these susceptibility genes. The NRG-1 is a member of neuregulin family that acts on the EGFR family of receptors. The NRG-1 is produced in numerous isoforms by alternative splicing, which allows it to perform a wide variety of functions such as regulation of neural development, neurotransmission, and synaptic plasticity through activation of the ErbB receptors. Binding of NRG-1 to ErbB enhanced the phosphorylation of both the ErbB receptors and CREB (Ozaki et al., 2004). The transcription factor early growth response 3 (Egr3) is an immediate-early growth response gene, which is transcriptionally induced by NRG1, and the induction of Egr3 in response to NRG1 is associated with the activation of ERK1/2 and possibly CREB phosphorylation (Herndon et al., 2014).

Collectively, as we previously reviewed (Zheng et al., 2012), all these genes are linked to Akt: (i) DISC1 is a protein involved in the modulation of neuronal proliferation, differentiation, and neuronal outgrowth. Dysregulated expression of DISC1 may predispose individuals to the development of schizophrenia and other psychiatric conditions. DISCI interacts and promotes the activation of Akt (Dahoun et al., 2017); (ii) the PI3K/Akt signaling pathway mediates the neuroprotective effect of NRG1 (Papaleo et al., 2016); (iii) Dysbindin is another protein highly expressed in neural tissues. Dysbindin-1 showed protective effect in neurons against apoptosis through activating the PI3K/Akt signaling in cortical neurons (Zheng et al., 2012). Cumulatively, these findings link the biological effects of these genes with PI3K/Akt signaling pathway. Considering that CREB is one of the downstream key effector modules of the PI3K/Akt signaling pathway (Sun et al., 1994; Li et al., 2011), it is plausible that there is a cross-talk between susceptibility genes and Akt/CREB pathway, although we cannot exclude the possibility that they work independently.

Besides the susceptibility genes listed earlier, there are many other risk-genes that have been identified in schizophrenia (Kato, 2015). It is hypothesized that these susceptibility and/or risk-genes trigger a clinically measurable outcome. The CREB may act as a possible signaling molecule link in the pathophysiology process triggered by the various susceptibility genes. Future human genetic studies will be crucial for shedding light on this concept.

### Role of CREB in Therapy of Schizophrenia

Many studies addressed the expression and activity of CREB, a transcription factor regulated by cAMP/PKA, in animal models upon treatment with antipsychotic drugs. The CREB mRNA levels are not regulated by haloperidol in striatum of rats, while haloperidol induces a significant phosphorylation of CREB in striatum, indicating that CREB is transcriptionally active in response to haloperidol (Konradi et al., 1993). In amphetamine-treated rats, haloperidol also induced a distinct immediate early gene (such as c-fos, c-jun) expression and CREB phosphorylation, and these neurochemical changes are associated with behavioral plasticity (Hsieh et al., 2002). In contrast to these findings, the drug olanzapine increased protein levels of CREB and BDNF in the prefrontal cortex, hippocampus, and striatum of adult Wistar rats (Reus et al., 2012). *In vitro* studies also support this conclusion. In SY5Y neuroblastoma cells, amisulpride, an atypical antipsychotic drug, stimulated neurite outgrowth by regulating D2R-mediated β-arrestin 2 signaling, and increased levels of CREB phosphorylation, with BDNF potentially involved in this process (Park et al., 2011). Similarly, olanzapine treatment increased basal BDNF gene in SY5Y cells. It is supposed that olanzapine activated PKA, PI3K, PKC, and CaMKII signaling pathways and subsequently upregulated BDNF gene transcription via activating CREB (Lee et al., 2010). A 4-week treatment with both olanzapine and lithium in rats led to a 1.4-fold increase of the levels of CREB and BDNF in the dentate gyrus and hippocampal area CA1. These observations support that the activation of CREB and upregulation of BDNF may underlie the neurological actions of olanzapine and lithium (Hammonds and Shim, 2009).

We would like to propose that various antipsychotic drugs may have different roles on CREB phosphorylation in neurons in both culture and *in vivo* situations. It was reported that haloperidol and eticlopride, selective D2R antagonists, stimulated the phosphorylation of CREB in the dorsal striatum. In contrast, clozapine reduced CREB phosphorylation, indicating that haloperidol and clozapine induce distinct patterns of CREB phosphorylation in the dorsal striatum (Pozzi et al., 2003). On the other hand, neurons at different maturation stages may have distinct phenotypes regarding the phosphorylation of CREB in response to the same antipsychotic drugs. For example, low concentrations (50 nM) of haloperidol and risperidone stimulate the phosphorylation of both ERK and CREB in hippocampal neuron cultures after 25 days *in vitro*, but not at 10 days (Yang et al., 2004). These different experimental observations may reflect developmental changes in the ratios of expression of the different dopamine receptors in neurons (Yang et al., 2004).

## Conclusion and Perspectives

Current studies suggest that CREB is a key integrator of diverse physiological processes in the CNS, including neurotransmission, neurodevelopment, neuronal survival, synaptic plasticity, and memory. Importantly, dysregulation of the CREB signaling has been implicated in a number of disorders in the CNS. Among these disorders, the relevance of CREB to the pathogenesis of schizophrenia has been most intensively investigated. The roles of CREB in the pathology of schizophrenia are depicted schematically in **Figure 3**. Dopamine, antipsychotic drugs, growth factors, and susceptibility genes could activate CREB and its downstream target BDNF via different pathways. The deficit of CREB/BDNF signaling impairs neurodevelopment and is implicated in the development of schizophrenia.

Given the important role of CREB activation in the CNS, it is reasonable to propose that activation of CREB signaling would have beneficial effects in schizophrenia therapy. However, sustained CREB activation also causes deleterious consequences. Hence, keeping a balance on CREB activation is a reasonable strategy in schizophrenia therapy. Pharmacological interventions, using transcriptional repressors, may serve this purpose. On the other hand, besides Ser133, CREB is also phosphorylated at many other sites. Investigating the relationship between these phosphorylation sites and schizophrenia may lead to novel pharmaceutical approaches. Among these sites, Ser129 and Ser 142 are close to Ser133, and it is tempting to suggest that these two sites will affect the transcription activity or binding of CREB to CRE. Moreover, the activity of CREB is modulated by additional signaling pathways, which due to antagonistic action may be manipulated for schizophrenia therapy. The signaling pathways involved in schizophrenia are complex. Both CREB and BDNF are key molecules implicated in the regulation of mood. Additional basic and clinical research is needed to further identify the specific role of the CREB in the pathology of schizophrenia, as this could shed new light on the effective management of this psychiatric disorder. Furthermore, CREB regulates the transcription of multiple genes, and extensive efforts are required to identify their involvement in the pathogenesis of schizophrenia and their myriad of combinatory effects.

The CREB-mutant mice, especially conditional knockout mice, represent a useful tool for examining its role in mediating neuropsychiatric behaviors. The CREB^αΔ^ mutant mice is homozygous mice with a targeted mutation in CREB; the α and Δ isoforms of CREB are knocked down in this animal model, whereas the CREB β variant is upregulated (Blendy et al., 1996). The functional consequence of this mutation is a reduction of 90% in CRE binding and activity (Walters and Blendy, 2001). Currently, findings with CREB^αΔ^ mutant mice support that enhanced CREB expression and phosphorylation promote neurodevelopment (Malberg and Blendy, 2005), while downregulation of CREB activity in mice would impair neurogenesis (Nakagawa et al., 2002). CREB-mutant mice have been used for the studies on the mechanisms of depression and the therapeutic effects of antidepressants (Conti et al., 2002; Gur et al., 2007). We propose that this animal model is useful for studying the relationship between CREB activation and the etiology of schizophrenia, and it may help to investigate the responses of mice to antipsychotics. However, there are a few disadvantages with this animal model, as CREB is widely expressed in the brain and CREB deficiency in this animal model is not strictly located to a specific neuronal populations (Gass and Riva, 2007). On the other hand, CREB deficiency is occurring throughout the whole body and during the initial fetal development in this animal model, which might cause developmental deficits and/or possible compensatory effects for the loss of CREB (Gass and Riva, 2007).

In summary, targeting CREB proteins might promote synaptic plasticity and neurodevelopment in CNS, which can be beneficial or delay the development of schizophrenia. However, we also would like to point out that the increased risk of suicide should be take into consideration when targeting CREB, as data from human postmortem suggest that the numbers of phosphorylated CREB-positive cells were increased in amygdala in subjects, who had died by suicide, while lithium could significantly decrease the phosphorylation of CREB levels in the same region (Young et al., 2004). Hence, the phosphorylation level of CREB in the brain sections highly related with mood disorders should be maintained at a certain level, namely neither too high, nor too low. Whether or not this strategy can be applied for the treatment of schizophrenia will depend upon further research characterizing the importance of CREB in the pathological processes of schizophrenia.

## Author Contributions

HW initiated the research topic and discussed the literature and wrote the draft manuscript. WZ performed the conceptional design and writing of the final manuscript. JX wrote some part of the review. PL and RQ contributed constructive suggestions and extensive language editing.

## Conflict of Interest Statement

The authors declare that the research was conducted in the absence of any commercial or financial relationships that could be construed as a potential conflict of interest.
